# Synergistic effect of carbon nanoparticles with mild salinity for improving chemical composition and antioxidant activities of radish sprouts

**DOI:** 10.3389/fpls.2023.1158031

**Published:** 2023-05-31

**Authors:** Riyadh F. Halawani, Hamada AbdElgawad, Fahed A. Aloufi, Mansour A. Balkhyour, Ahlem Zrig, Abdelrahim H.A. Hassan

**Affiliations:** ^1^ Department of Environment, Faculty of Environmental Sciences, King Abdulaziz University, Jeddah, Saudi Arabia; ^2^ Botany and Microbiology Department, Faculty of Science, Beni-Suef University, Beni-Suef, Egypt; ^3^ Higher Institute of Preparatory Studies in Biology and Geology, University of Carthage, Tunis, Tunisia; ^4^ Laboratory of Engineering Processes and Industrial Systems, Chemical Engineering Department, National School of Engineers of Gabes, University of Gabes, Gabès, Tunisia; ^5^ School of Biotechnology, Nile University, Giza, Egypt; ^6^ Department of Food Safety and Technology, Faculty of Veterinary Medicine, Beni-Suef University, Beni-Suef, Egypt

**Keywords:** carbon nanoparticles, mild salinity, radish sprouts, anthocyanin, polyamines, proline

## Abstract

The demand for healthy foods with high functional value has progressively increased. Carbon nanoparticles (CNPs) have a promising application in agriculture including the enhancement of plant growth. However, there are few studies on the interactive effects of CNPs and mild salinity on radish seed sprouting. To this end, the effect of radish seed priming with 80mM CNPs on biomass, anthocyanin, proline and polyamine metabolism, and antioxidant defense system under mild salinity growth condition (25 mM NaCl). The results indicated that seed nanopriming with CNPs along with mild salinity stress enhanced radish seed sprouting and its antioxidant capacity. Priming boosted the antioxidant capacity by increasing antioxidant metabolites such as (polyphenols, flavonoids, polyamines, anthocyanin, and proline). To understand the bases of these increases, precursors and key biosynthetic enzymes of anthocyanin [phenylalanine, cinnamic acid, coumaric acid, naringenin, phenylalanine ammonia lyase, chalcone synthase (CHS), cinnamate-4-hydroxylase (C4H) and 4-coumarate: CoA ligase (4CL)], proline [pyrroline-5-carboxylate synthase (P5CS), proline dehydrogenase (PRODH), Sucrose, Sucrose P synthase, invertase) and polyamines [putrescine, spermine, spermidine, total polyamines, arginine decarboxylase, orinthnine decarboxylase, S-adenosyl-L-methionine decarboxylase, spermidine synthase, spermine synthase] were analyzed. In conclusion, seed priming with CNPs has the potential to further stimulate mild salinity-induced bioactive compound accumulation in radish sprouts.

## 1 Introduction

Sprouts, the immature seedlings formed by seed germination and one of the most nutrient-dense plant sources, have drawn a lot of interest because of the important phytochemicals they contain. They are abundant in vitamins, amino acids, fatty acids, and antioxidants, for example, which support their functions as antioxidants and anticancer agents (Bachiega et al., 2016; Almuhayawi et al., 2021). Besides, sprouts have comparatively fewer antinutrients compared to their mature plants. (Popova and Mihaylova, 2019).

According to phylogenetic analyses of the *Brassicaceae* family, the radish (*Raphanus sativus* L.) is a plant species that belongs to the genus *Raphanus* and is part of the Rapa/Oleacera lineage (Gamba et al., 2021). One of the key properties of radish is its phytochemical composition. Anthocyanin pigments give radish roots their red color, and its strong ability to synthesize isothiocyanates gives them their distinctive flavor, which is highly popular in countries like Japan, the Philippines, and Hawai (Gupta et al., 2003; Nishio, 2017).

Radish extracts have been employed in treating several health disorders (Manivannan et al., 2019). The possible use of radish as a source of bioactive compounds with clinical and pharmaceutical implications in diseases such as high blood pressure, and cardiac disorders and as an antioxidant and antimicrobial agent has become a field of interest for several studies and the therapeutic industry (Lim, 2012; Manivannan et al., 2019). Therefore, improving the nutritional and phytochemical composition of radish will maximize the potential use of radish in human nutrition and biological and pharmaceutical applications.

Any external abiotic (salinity, heat, water, etc.) or biotic (herbivore) constraint that slows down photosynthesis and makes it harder for plants to convert energy into biomass is referred to as stress (Grime, 1977). Global agriculture is being faced with numerous issues, such as the fact that crop productivity is not rising at the same rate as global food consumption. At high levels, abiotic stressors such as salinity, drought, cold, and heat negatively influence the endurance, growth, and yield of staple food crops up to 70% (Ahmad and Sharma, 2008; Parihar et al., 2015). Salinity is defined as the unfavorable impact of excess minerals such as Na+ and/or Cl- on plants (Munns, 2005). Several studies have investigated the effect of salinity on plants (Abdelaal et al., 2020; Hassan et al., 2020; Osman et al., 2021).

Although extreme abiotic stresses, like salt stress, have a negative impact on plant growth, mild stresses can be purposefully induced to increase the antioxidant content of the plant’s edible section and promote plant tolerance to stressful circumstances (Kim et al., 2008; Cogo et al., 2011). The prevention of several diseases and the improvement of human health are positively correlated with the consumption of fruit and vegetables rich in antioxidant compounds; for this reason, efforts to improve the nutritional and functional quality of foods during plant cultivation, a strategy known as biofortification, are of great interest (Zhu et al., 2013; Galli et al., 2016).

Nanotechnology is a rapidly developing science with a promising future in agriculture. Numerous publications claim that nanotechnology has received significant attention in the domains of agriculture and the environment. Numerous nanomaterials have been created for use in agriculture, including innovative approaches to soil and water remediation, inducers of plant germination and growth, and nanopesticides and nanofertilizers that can be used in smaller amounts while still enhancing food production and quality (Scott et al., 2018; Do Espirito Santo Pereira et al., 2021). On the other hand, recent studies reported that some nanoparticles have adverse impact on plants, including the retard of germination and phytotoxicity of plant seedlings (Pelegrino et al., 2020). However, others can act as stimulants, improving plant growth by acting in cellular signaling pathways (Acharya et al., 2020). This impact depends on the physical-chemical characteristics of nanoparticles, such as size, and zeta potential, which determine the biological responses (Pérez-De-Luque, 2017).

These features play important roles in the absorption and transport of nanoparticles in plants (Hu et al., 2020). The nanoparticle’s surface charge is also a decisive factor. Nanoparticles with either a positive or negative charge can be absorbed by the leaves and transported to the roots. Only negatively charged nanoparticles, however, are absorbed by the roots because positively charged particles cause the creation of mucilage, which stops plants from absorbing positively charges particles (Spielman-Sun et al., 2019).

Carbon nanoparticles (CNPs) have explored many potential applications in agricultural production (Joshi et al., 2018). CNPs applied to plants appear to pierce into the root epidermal cells and accumulate in the tissues (Wild and Jones, 2009), which could modulate cellular function. Water-soluble single-wall CNPs were found to improve the root and shoot growth of legume seeds; this improvement was mostly related to an increase in water uptake (Wild and Jones, 2009; Wang et al., 2012). Growth enhancement by multi-walled CNPs has also been stated for other plant species such as mustard (*Brassica juncea*) and some cereals (Mondal et al., 2011; Wang et al., 2012; Tiwari et al., 2014).

Nano-priming can be employed to protect plant seeds during storage, enhance germination, boost plant growth, and raise crop resistance to biotic or abiotic stress conditions. This can help to lower the amounts of pesticides and fertilizers needed. (Malik et al., 2020). Studies revealed that seed nano-priming can activate several genes, particularly those linked to plant stress tolerance, during germination (Mahakham et al., 2017; An et al., 2020). Seed nano-priming also can be applied for seed protection, as several types of nanoparticles have antimicrobial effects and also can be loaded by antimicrobial agents (Abbasi Khalaki et al., 2021). Additionally, the biofortification of seeds by nano-priming can be employed to encourage an improvement in food quality and production (De La Torre-Roche et al., 2020; Malik et al., 2020).

Although many studies investigated the impact of mild salinity on plant production and biological activities, as well as nano-priming of plant seeds recently attracted the attention of researchers in the agriculture discipline, to the best of our knowledge, no previous research works investigated the interactive impact of seed priming with CNPs along with mild salinity. Therefore, the current study was carried out to identify the effect of radish seed priming with CNPs on the biomass, pigment production, anthocyanin metabolism, proline metabolism, polyamine metabolism, and antioxidant capacity, metabolites and enzymes of radish sprouts under mild salinity growth condition. We hypothesize that seed nanopriming will improve mild salinity-induced bioactive compound accumulations in radish sprouts.

## 2 Materials and methods

### 2.1 Sprout production

Seeds of radish species were surface sterilized by submerging in a 50% (v/v) solution of commercial sodium hypochlorite (2.5 g 100 g^-1^) for 10 min and rinsed once with sterile deionized water. The seeds were divided into four groups: a control group, salt treated group, CNPs-treated group and CNPs with salinity-treated group. 50 radish seeds were immersed in 20 mL of solution with 80 mM of CNPs (group 2). After 12 h of imbibition, the treated (group 2) and untreated (group 1, control) seeds were germinated on trays containing vermiculite. Germinated seeds were watered using Milli-Q water, two times per week. Hoagland nutrient solution was supplied once at the start of the experiment to nourish the seedlings. To induce mild salinity, 25 mM NaCl was always added to the nutritive solution. This was according to a preliminary experiment with a range of NaCl concentrations (5 to 50 mM). We selected the most effective concentration that induced bioactive compound accumulation withought severe inhibition in radish sprout growth. Trays of germinated seed were moistened with 10 mL of 25 mM NaCl solution (group 4) or DI water (group 3). All treated sprouts were grown in a climate-controlled chamber at 21/18°C, over a 16/8 h day/night photoperiod (150 µmol PAR m^−2^ s^−1^, with 60% humidity). After ten days of growth, sprouts from each tray were weighed to determine their fresh weight and then stored at 80°C for a further biochemical analysis. Fifteen plants from each tray were mixed and used as biological duplicates for each measurement. Each experiment was repeated three times.

### 2.2 Carbon nanoparticles (CNPs)

Water-dispersible CNPs, composed of 63% of C, 34% of O, 1.6% of H 1.4% of N, were obtained from Vulpes Inc. (St. Louis, MO, USA). According to the purchasing source, the CNPs had a size range of 20–130 nm with negative charges on surfaces (zeta potential −67.6 mV). The specific surface area of 35–50 m^2^ g^−1^ and porosity ranges of tested CNPs were 7–11%.

### 2.3 Determination of total antioxidant activity, phenols, flavonoids, and antioxidant metabolites and enzymes

The antioxidant activity of radish sprouts was carried *in vitro* through ferric reducing antioxidant power (FRAP) method according to (Al Jaouni et al., 2018; Hamed et al., 2019). Approximately 0.2 g of each radish sprout samples was diluted with 80% ethanol and centrifuged for twenty min at 14,000 rpm. Then 0.1 mL of diluted extract was used to determine the antioxidant activity after mixing with 0.25 mL of FRAP reagent [mixing FeCl_3_ (20 mM) in acetate buffer (0.25 M, pH 3.6) at room temperature] (El-Soud et al., 2013). The absorbance was measured at 517 nm. Additionally, the activity of peroxidase (POX) and catalase (CAT) enzymes was determined in each frozen radish sprout group in 1 mL of 50 mM MES/KOH (pH 6.0) extraction buffer after 10 min of incubation (Sinha et al., 2015; Hamed et al., 2017).

Chromatographic techniques, such as HPLC and GC/MS, were employed to determine the levels of polyphenols, flavonoids, and vitamins in treated and control radish sprouts (Hamad et al., 2015). The metabolites were identified through matching the standard mixture to the relative retention time of each metabolite from each sample. Using the peak area of the corresponding standard, the concentration of each metabolite was calculated.

To determine polyphenols and flavonoids using HPLC, 50 mg of freeze-dried sprouts were mixed in 4:1 v/v acetone–water solution. The Shimadzu HPLC system, equipped with a Lichrosorb Si-60, 7 µm, 3 × 150 mm column, diode array detector (SCL-10 AVP, Japan) was used. Water-formic acid 90:10 (v/v) and acetonitrile/water/formic acid 85:10:5 (v/v/v) at 0.8 mL/min (flow rate) were employed as a mobile phase and the internal standard was 3,5-dichloro-4-hydroxybenzoic. Using the peak area of the corresponding standard, the concentration of each metabolite was calculated.

To estimate rubisco activity, frozen sprouts discs were extracted using 1 mL cm^-2^ of 100 mM Tricine-NaOH, pH 8, 5 mM MgCl_2_, 1 mM EDTA, 5% PVP-40, 6% PEG-4000, 5 mM DTT, 1 mM phenylmethylsulfonyl fluoride, and 10 M leupeptin in Ten Broeck glass homogenizers. Assays were carried out at 30°C either immediately upon extraction or following a 20-second, 10,000-g centrifugation period. On transparent 96-well plates, 0.02 mL of sprout extract was added to the assay mix for a final volume of 0.2 mL to measure the first rubisco activity. The assay mixture included 0.96 U enolase, 0.75 U dPGM, 0.2 mM 2,3-bisPGA, 2 mM ADP, and 0.5 mM RuBP. It also contained 1.85 U pyruvate kinase, 2.33 U lactate dehydrogenase, and 1.85 U pyruvate kinase. Sprout extracts were incubated in the test mixture without RuBP to completely carbamylate rubisco in order to assess overall activity (Carmo-Silva and Salvucci, 2013). Immediately following the addition of the sprout extract to the assay mix containing 1 mM RuBP (initial), or after 3 min incubation in the assay mix prior to the addition of RuBP, a Synergy HT (Bio-Tek, Denkendorf, Germany) plate reader was used to measure the rate of decrease in absorbance at 340 nm during the first 1-2 min of the assay (total). In certain experiments, assays were carried out in microcuvettes, and a UV-Vis spectrophotometer was used to measure the absorbance at 340 nm (Varian, Cary Bio100). The assay volume for these reactions was 0.4 mL overall, and the sprout extract volume was 0.04 mL.

### 2.4 Determination of vitamin and pigment contents

To determine the contents of vitamins and pigments in radish sprouts, UV and/or fluorescence detectors were used for the separation and detection processes according to (Abdelgawad et al., 2015). Ascorbate was extracted in 1 mL meta-phosphoric acid 6% (w/v) at 4°C and was separated by a reversed-phase HPLC coupled with UV detector (100 mm × 4.6 mm Polaris C18-A, 3 lm particle size; 40°C, isocratic flow rate: 1 mL min^−1^, elution buffer: 2 mM KCl, pH 2.5 with *O*-phosphoric acid). Tocopherols were separated on Particil Pac 5 µm column material (length 250 mm, i.d. 4.6 mm) and quantified by HPLC (Shimadzu’s Hertogenbosch, normal phase conditions), coupled with fluorometric detector (excitation at 290 nm and emission at 330 nm). The frozen radish sprout samples were homogenized in acetone through a MagNALyser (Roche, Vilvoorde, Belgium, 1 min, 7000 rpm), then centrifuged for 20 min at 14000*g* at 4°C. The supernatant was taken and filtered through Acrodisc GHP filter (0.45 μm 13 mm). Afterward, the solution was analyzed by using HPLC (Shimadzu SIL10-ADvp, reversed-phase, at 4°C) (Abdelgawad et al., 2015). Carotenoid separation was done on a silica-based C18 column (Waters Spherisorb, 5 µm ODS1, 4.6 × 250 mm) using two types of solvents; consisting of acetonitrile: methanol: water solvent as 81:9:10 and methanol: ethyl acetate solvent as 68:32. The extraction of Chlorophyll *a* and b, alpha-carotene and beta-carotene were measured through a diode-array detector (Shimadzu SPD-M10Avp) at different four wavelengths (420, 440, 462, and 462 nm, respectively).

### 2.5 Measurements of polyamines and their metabolic enzymes

Polyamines were extracted in perchloric acid (Bregoli et al., 2002). After centrifugation for 40 min at 15,000g, extracts were derivatized with dansyl-Cl. Then hydrolyzation at 115°C in HCl (6 N) was added for the formation of conjugated polyamines. The reverse phase of HPLC (Shimadzu SIL10-Advp; and separation on C18 column) was applied. The activities of polyamine biosynthetic enzymes, plant shoot and roots were homogenized (120 mM KPO4, pH 7.4) then they were centrifuged at 27,000 RPM for 25 min. Arginine decarboxylase (ADC) and ornithine decarboxylase (ODC) enzymes activities were measured by measuring the labeled CO_2_ (Birecka et al., 1985). Spermidine synthase (SpdS) activity was assayed in a reaction mixture (pH 8.0, 110 mM Tris-HCl) (Yoon et al., 2000). The production of 5′-deoxy-5′-methylthioadenosyne was quantified by using a fluorescence detection method (a reverse phase HPLC). However, activity of spermine synthase (SpmS) was measured by following the methylthioadenosine production (Lauren Cason et al., 2003).

### 2.6 Extraction and estimation of anthocyanin and metabolic enzymes

Total anthocyanins were extracted by homogenizing in 15 mL of acidified methanol (99:1, v/v). After extraction, the homogenate was incubated at 27°C for a day in the dark. Extracts were centrifuged at 4000×g for 10 min. The anthocyanin content of the extract was quantified by measuring its absorbance at 550 nm. PAL enzymes were extracted (0.2 M of Na-borate buffer at pH 8.8). The activity was determined by measuring the production of trans-cinnamic acid (290 nm). The activity of 4CL (4-coumarate: coenzyme A ligase) was monitored by estimating the increase in p-coumarate (333 nm). Fresh samples were extracted in Tris-HCL buffer (50 mM, pH 8.9). The activity was estimated as the increase in 4-hydrox- y-trans-cinnamic acid (340 nm). One unit of 4CL activity equal to the production of 1.0 nmol hydroxy-trans-cinnamic acid per min (Balkhyour et al., 2021).

### 2.7 Determination of proline metabolism

Proline metabolites were extracted in 2 ml of 80% ethanol, spiked with norvaline as internal standard. Proline contents were determined by using a Waters Acquity UPLC-tqd system equipped with a BEH amide 2.1 × 50 column.The activities of key enzymes involved in proline metabolism, i.e., pyrroline-5- carboxylate synthetase (P5CS) and proline dehydrogenase (ProDH) were extracted in Tris-HCl buffer (50 mM, pH 7.4) (Shabbaj et al., 2022). The activities of enzymes were measured by monitoring the reduction of NADH at A340 and production γ-glutamyl hydroxamate at A535, the reduction of 2,6-dichloroindophenol at A600 (Temple et al., 1996).

### 2.8 Statistical analysis

The statistical analysis was applied using the SPSS statistical package (SPSS Inc., Chicago, IL, USA). One-way Analysis of Variance was conducted to all data and Tukey’s Test (*P<0.05*) was applied as the *post-hoc* test for separation of means. Each experiment was replicated at least three times (n ≥3).

## 3 Results

### 3.1 Effect of CNPs priming under mild salinity on radish sprout biomass

**Figure 1:**
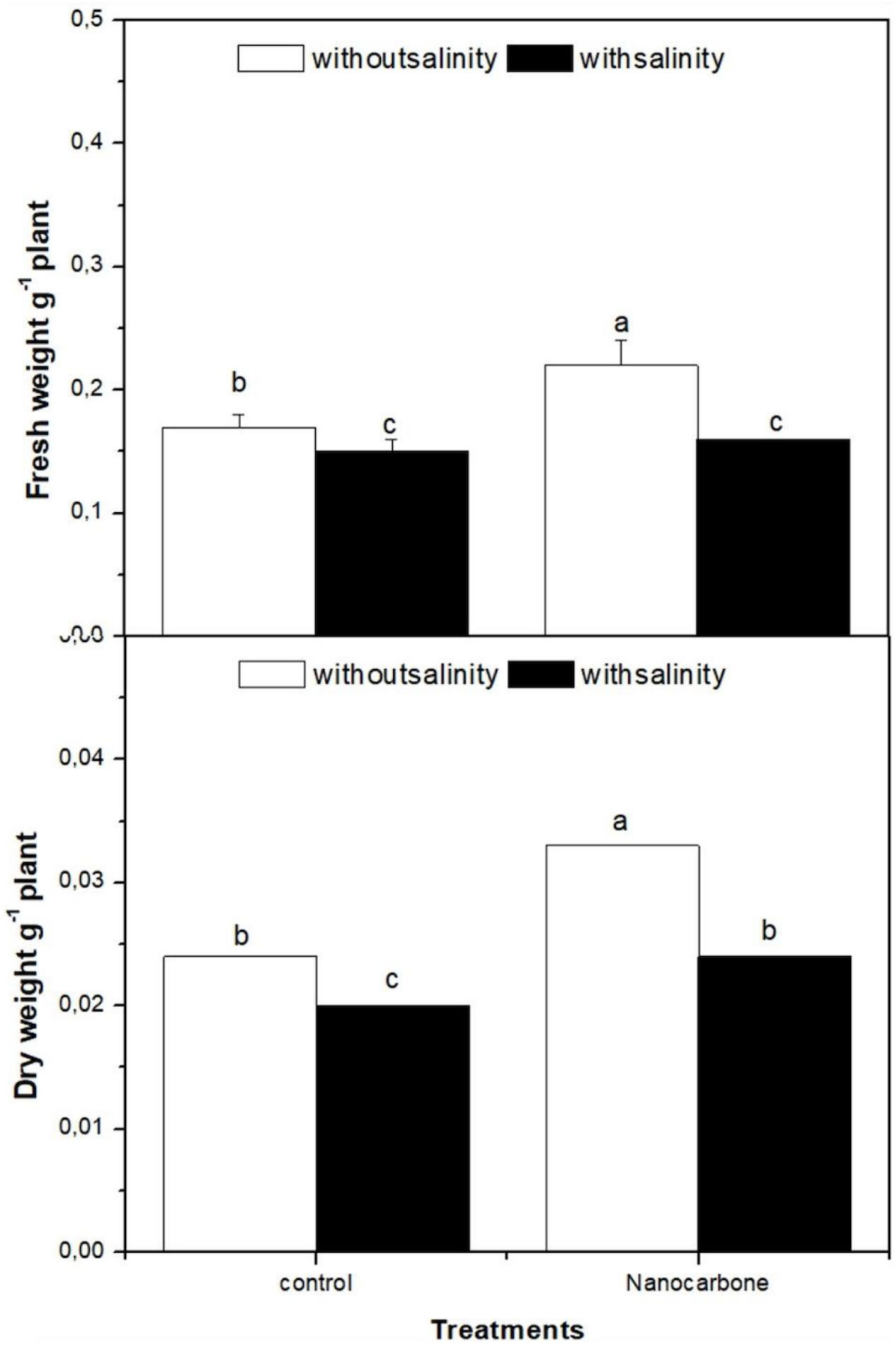
Effect of seed priming with carbon nanoparticles (CNPs) either alone or in combination with mild salinity on fresh weight (g FW), and dry weight (g DW) of radish sprouts. Data are represented by the means of three replicates and error bars represent standard error. Different small letters on the bars indicate significant differences between means at p<0.05.

The effect of mild salinity and/or seed priming in CNPs on the biomass of radish sprouts as compared to control groups was investigated (**Figure 1**). Apparently, salt treatment significantly decreased the fresh and dry weights of radish sprouts in comparison with the control group. On the other hand, seed priming with CNPs mitigated the harmful effect of mild salinity on radish sprout growth. As in the case of CNPs treatment of seeds, the fresh and dry weights of radish sprouts were approximately duplicated as compared with the control in both salinity-treated and untreated groups.

### 3.2 Effect of CNPs priming under mild salinity on pigment contents of radish sprouts

The data illustrated in **Table 1** show the levels of rubisco (a photosynthesis enzyme) and pigments in radish sprouts primed with CNPs and grown under a mild salinity environment in comparison with the control. It was found that priming in CNPs besides salinity has a synergistic effect on the levels of pigments. Sprouts primed in CNPs and grown in mild salt had the significantly highest (p<0.05) level of chlorophyll a + b (5.53 ± 0.62) followed by CNPs combined with mild salinity stress, while the lowest value (1.90 ± 0.21) was detected in control sprouts without CNPs priming or salinity. A similar scenario was noticeable in the case of rubisco. Additionally, the levels of individual pigments including alpha-carotene, beta-carotene, lutein, and beta-cryptoxanthin were significantly higher (p<0.05) in the case of sprouts treated with both CNPs and mild salinity. On the other hand, control sprouts that were not exposed to either CNPs or salinity had the significantly lowest values of most measured individual sprouts. CNPs priming and salinity environment increased chlorophyl a + b level, rubisco, alpha carotene, and beta-cryptoxanthin by about 3 folds as compared to the control group. Rubisco was the most abundant pigment in radish sprouts, followed by chlorophyl a + b, while beta-cryptoxanthin was the lowest one.

**Table 1:** Effect of seed priming with carbon nanoparticles (CNPs) either alone or in combination with mild salinity on pigment contents in radish sprouts.

Pigments (mg/g FW)	Without CNPs priming		With CNPs priming	
	Control	Salinity	Control	Salinity
**Chlorophyll a + b**	4.39 ± 0.26c	4.06 ± 0.17d	5.53 ± 0.62a	4.74 ± 0.99b
**Rubisco**	6.89 ± 0.33b	6.13 ± 0.76b	8.21 ± 0.75a	6.96 ± 0.54b
**Alpha-carotene**	0.27 ± 0.02c	0.38 ± 0.06b	0.31 ± 0.00b	0.41 ± 0.41a
**Beta-carotene**	0.11 ± 0.01c	0.19 ± 0.03b	0.11 ± 0.01c	0.21 ± 0.03a
**Lutein**	0.18 ± 0.02c	0.19 ± 0.06c	0.23 ± 0.02b	0.28 ± 0.02a
**Beta-cryptoxanthin**	0.06 ± 0.00c	0.08 ± 0.02b	0.08 ± 0.01b	0.12 ± 0.01a

Data are represented by means of three replicates ± standard errors. Different small letters within rows indicate significant differences between means at p<0.05.

### 3.3 Effect of CNPs priming under mild salinity on anthocyanin metabolism of radish sprouts

Also, the anthocyanin metabolism was monitored in radish sprouts with or without exposure to CNPs priming and grown under normal salt or mild salt concentrations (**Table 2**). Radish sprouts exposed to both CNPs priming and salinity displayed the significantly highest levels (p<0.05) of all measured parameters of anthocyanin metabolism except phenylalanine ammonia lyase. Two-fold increase or more were recorded in anthocyanin, phenylalanine, cinnamic acid, coumaric acid, naringenin, chalcone synthase CHS, C4H and 4CL in the case of double treatment. Naringenin was the most abundant parameter in radish sprouts followed by cinnamic acid, whereas 4CL was the lowest one.

**Table 2:** Effect of seed priming with carbon nanoparticles (CNPs) either alone or in combination with mild salinity on anthocyanin metabolism of radish sprouts.

Metabolites (Unit/mg prot. Min)	Without CNPs priming		With CNPs priming	
	Control	Salinity	Control	Salinity
**Anthocyanin**	0.51 ± 0.86b	0.56 ± 0.94b	0.45 ± 0.76b	1.12 ± 1.88a
**Phenylalanine**	0.60 ± 0.12b	1.02 ± 0.16ab	0.50 ± 0.10b	1.29 ± 0.25a
**Cinnamic acid**	3.39 ± 0.19c	5.44 ± 0.31b	2.20 ± 0.13c	7.06 ± 0.40a
**Coumaric acid**	1.50 ± 0.09b	1.65 ± 0.14b	1.33 ± 0.07c	3.30 ± 0.18a
**Naringenin**	3.49 ± 2.64c	4.20 ± 3.15b	3.35 ± 2.55c	8.35 ± 6.46a
**Phenylalanine ammonia lyase**	1.19 ± 0.88c	1.98 ± 1.43a	0.99 ± 0.73d	1.77 ± 1.32b
**Chalcone synthase (CHS)**	0.75 ± 0.58a	0.51 ± 0.43b	0.43 ± 0.34b	0.75 ± 0.59a
**Cinnamate-4-hydroxylase (C4H)**	1.47 ± 1.14b	1.64 ± 1.25b	1.31 ± 1.01b	3.16 ± 2.47a
**4-coumarate: CoA ligase (4CL)**	0.30 ± 0.25b	0.33 ± 0.28b	0.32 ± 0.26b	0.64 ± 0.52a

Data are represented by the means of three replicates ± standard errors. Different small letters within rows indicate significant differences between means at p<0.05.

### 3.4 Effect of CNPs priming under mild salinity on proline metabolism of radish sprouts

Additionally, in **Table 3** we summarized the effect of CNPs priming simultaneously with or without salinity on osmolytes metabolism including proline, P5CS, PRODH, sucrose, sucrose P synthase, and invertase in radish sprouts. CNPs priming induced significant increases in their levels (p<0.05), however, salinity mitigated the positive effect of CNPs priming as it significantly decreased the levels of P5CS, PRODH, sucrose P synthase, and invertase in radish sprouts, whereas sprouts grown without exposure to mild salt concentrations after CNPs priming of seeds showed the highest values in the case of P5CS, PRODH, sucrose P synthase and invertase (p<0.05).

**Table 3:** Effect of seed priming with carbon nanoparticles (CNPs) either alone or in combination with mild salinity on osmolytes metabolism of radish sprouts.

Metabolites	Without CNPs priming		With CNPs priming	
	Control	Salinity	Control	Salinity
**Proline (mg/gFW)**	6.16 ± 1.26b	3.80 ± 0.73c	7.48 ± 1.60a	6.65 ± 1.50a
**Pyrroline-5-carboxylate synthase (P5CS) (µmol/mg prot.min)**	3.78 ± 0.17b	3.51 ± 0.20c	5.08 ± 0.35a	4.80 ± 0.53ab
**Proline dehydrogenase (PRODH) (µmol/mg prot.min)**	8.88 ± 0.44b	7.74 ± 0.46c	10.17 ± 0.69a	8.20 ± 0.83b
**Sucrose (µg/g FW)**	2.86 ± 0.30c	3.72 ± 0.95b	4.64 ± 1.38ab	5.44 ± 0.56a
**Sucrose P synthase (µmol/mg prot.min)**	0.53 ± 0.01b	0.46 ± 0.01c	0.79 ± 0.02a	0.68 ± 0.02b
**Invertase (µmol/mg prot.min)**	1.08 ± 0.06b	0.95 ± 0.07c	2.14 ± 0.17a	1.94 ± 0.17b

Data are represented by the means of three replicates ± standard errors. Different small letters within rows indicate significant differences between means at p<0.05.

### 3.5 Effect of CNPs priming under mild salinity on polyamine metabolism of radish sprouts

Regarding the effect of both CNPs priming and salinity on polyamine metabolism in radish sprouts, polyamines including putrescine, spermine, spermidine, and total polyamines, as well as related enzymes including arginine decarboxylase, orinthnine decarboxylase, S-adenosyl-L-methionine decarboxylase, spermidine synthase and spermidine synthase were measured. Apparently, CNPs priming promoted the polyamine metabolism in radish sprouts, as well as it showed a synergistic effect with salinity exposure. This was clear in the values of both polyamine metabolites and enzymes, which were found to be significantly the highest (p<0.05) in radish sprouts exposed to both priming and salinity (**Table 4**).

**Table 4:** Effect of seed priming with carbon nanoparticles (CNPs) either alone or in combination with mild salinity on polyamine metabolism of radish sprouts.

Metabolites	Without CNPs priming		With CNPs priming	
	Control	Salinity	Control	Salinity
**Putrescine (Put) (pg/gFW)**	2192 ± 191c	2674 ± 173b	2837 ± 329b	3419. ± 298a
**Spermine (Spm) (pg/gFW)**	967 ± 80c	1145 ± 16.b	1195 ± 181b	1545.73 ± 43a
**Spermidine (pg/gFW)**	414 ± 34c	489 ± 3.70c	512.56 ± 77b	661.42 ± 18.a
**Total polyamines (pg/gFW)**	3574 ± 305c	4309 ± 191b	4545 ± 588b	5626 ± 318a
**Arginine decarboxylase (nmol/mg prot.min)**	13.85 ± 1.01c	15.10 ± 1.57b	16.57 ± 1.8b	23.10 ± 1.69a
**Orinthnine decarboxylase (µmol/mg prot.min)**	0.19 ± 0.02d	0.20 ± 0.03c	0.34 ± 0.04b	0.41 ± 0.04a
**S-adenosyl-L-methionine decarboxylase (nmol/mg prot.min)**	24.15 ± 1.75c	28.2 ± 3.19b	29.09 ± 3.11b	40.45 ± 2.91a
**Spermidine synthase (nmol/mg prot.min)**	25.13 ± 0.76c	29.49 ± 0.07c	30.80 ± 2.4b	40.0 ± 0.64a
**Spermine synthase (nmol/mg prot.min)**	13.74 ± 1.08c	16.21 ± 0.12b	16.98 ± 2.4b	21.92 ± 0.60a

Data are represented by the means of three replicates ± standard errors. Different small letters within rows indicate significant differences between means at p<0.05.

### 3.6 Effect of CNPs priming under mild salinity on antioxidant defence system in radish sprouts

**Figure 2:**
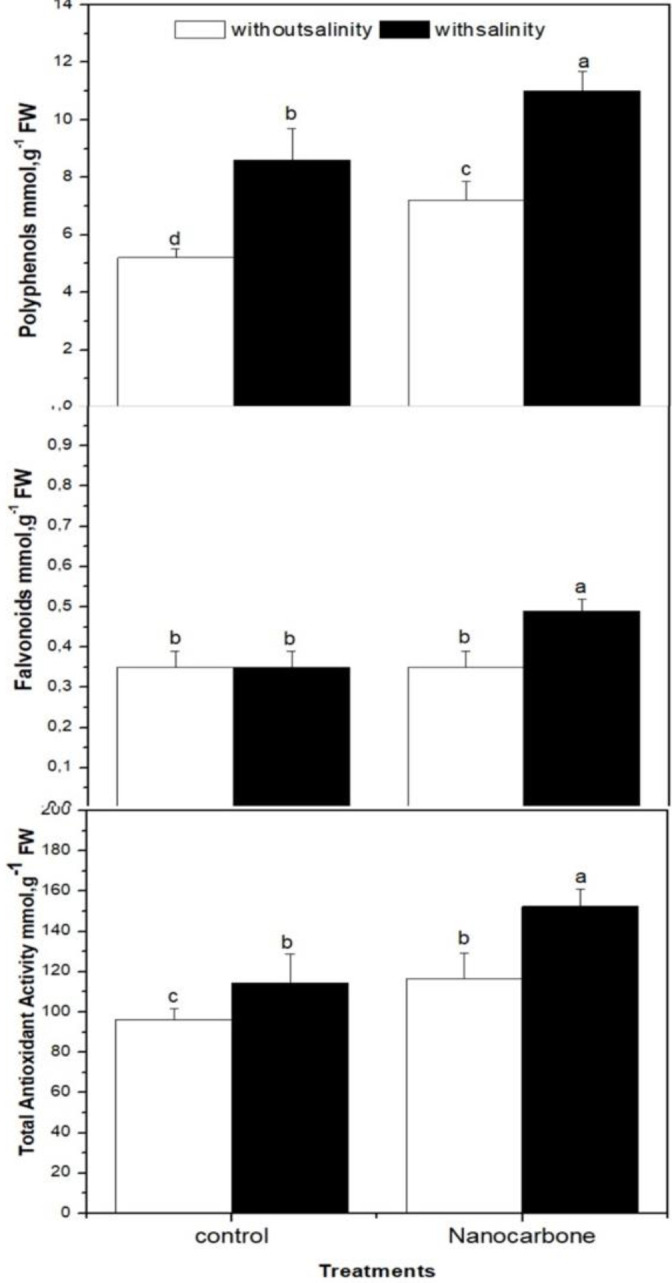
Effect of seed priming with carbon nanoparticles (CNPs) either alone or in combination with mild salinity on total antioxidant activity, and flavonoids and polyphenols contents of radish sprouts. Data are represented by the means of three replicates and error bars represent standard error. Different small letters on the bars indicate significant differences between means at p<0.05.

In addition, the effect of CNPs priming of seeds with or without mild salinity on the total antioxidant capacity, total flavonoids, and polyphenols of radish sprouts was studied in comparison with control groups (**Figure 2**). As a response to oxidative stress caused by salinity to radish sprouts, salt-treated sprouts revealed significantly higher values (p<0.05) of polyphenols and total antioxidant capacity than the control (untreated) group, whereas the values of flavonoids did not show an obvious change. Regarding the effect of CNPs priming of seeds on antioxidant metabolites and total antioxidant capacity, this study revealed that CNPs significantly enhanced (p<0.05) the production of flavonoids and polyphenols and improved the total antioxidant capacity of mild salinity ed radish sprouts. Moreover, CNPs treatment increased the polyphenols and total antioxidant capacity of radish sprouts that were not exposed to mild salinity, while the effect of CNPs on flavonoids in this group was minimal (**Figure 2**).

**Figure 3:**
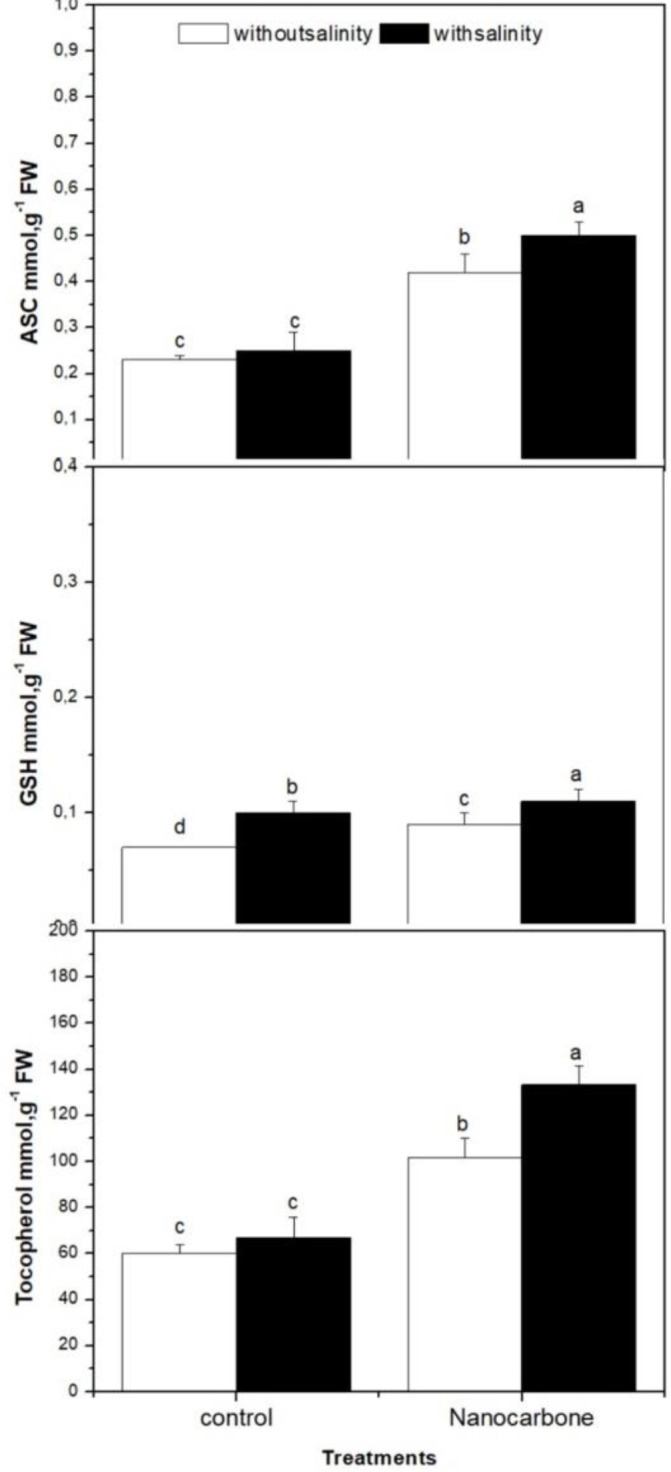
Effect of seed priming with carbon nanoparticles (CNPs) either alone or in combination with mild salinity on antioxidant metabolite production by radish sprouts. Data are represented by the means of three replicates and error bars represent standard error. Different small letters on the bars indicate significant differences between means at p<0.05.

The antioxidant metabolites namely ascorbates (ASC), glutathione (GSH) and tocopherols were also investigated in the case of four groups (control, salinity treated, nanotubes treated, both salinity and nanotubes treated) of radish sprouts (**Figure 3**). The impact of mild salinity on these antioxidant metabolites in the absence of CNPs treatment was weak, as appeared in group two in comparison with group one (control). On the other hand, seed priming with CNPs (group 3) significantly promoted the production of ASC, GSH, and tocopherols by radish sprouts than control groups, as well as mild salinity, showed a synergistic effect with nanotubes on the levels of these metabolites as it significantly increased their levels in the case of sprouts treated with both nanotubes and salinity (group 4) when compared with nanotubes treatment alone (group 3).

**Figure 4:**
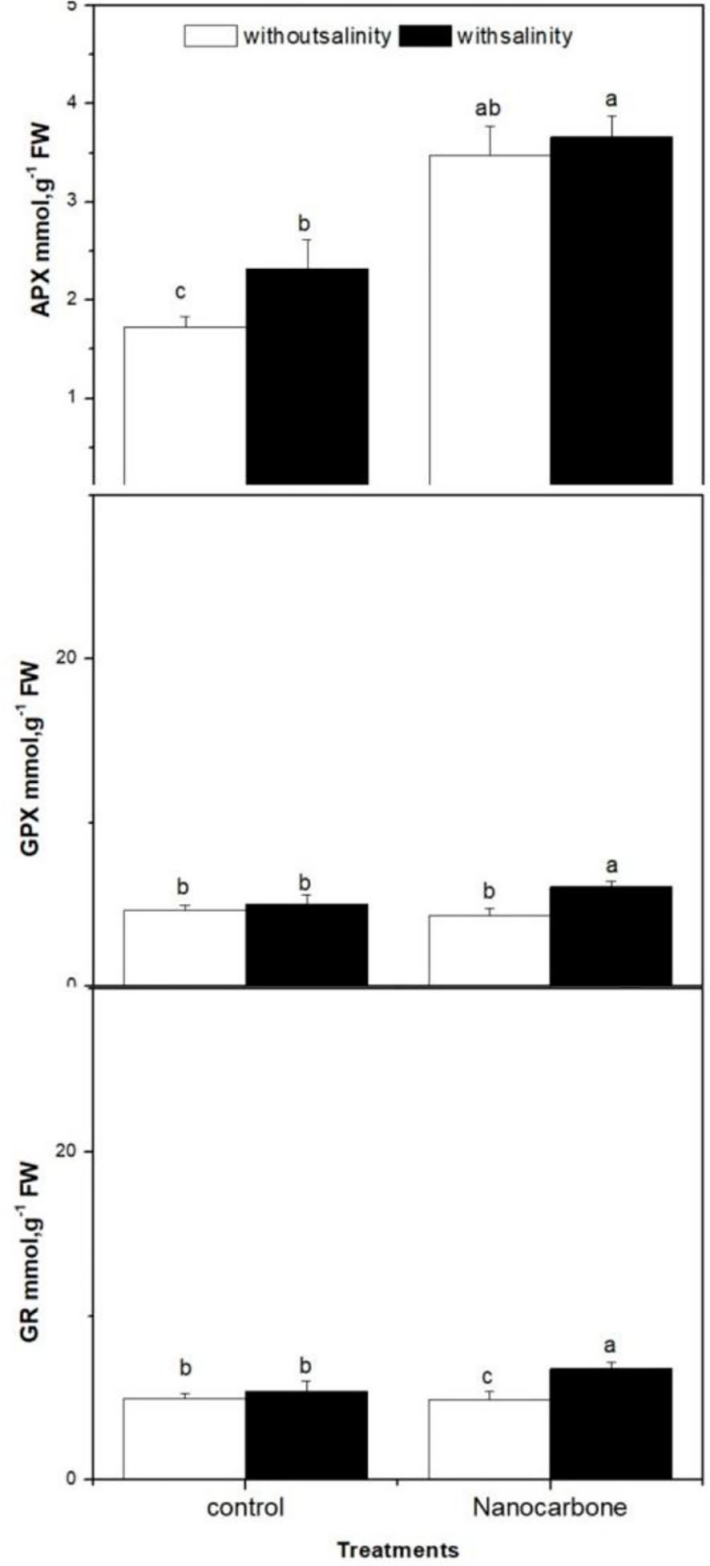
Effect of seed priming with CNPs either alone or in combination with mild salinity on the production of antioxidant enzymes (APX, GPX and GR) by radish sprouts. Data are represented by the means of three replicates and error bars represent standard error. Different small letters on the bars indicate significant differences between means at p<0.05.

The effect of mild salinity with or without seed priming with CNPs on the antioxidant enzymes (APX, GPX and GR) in radish sprouts was also monitored (**Figure 4**). The effect of salinity and CNPs individually on GPX and GR enzymes was minimal, as there were no significant differences between the control group (group 1), mild salinity-treated group (group 2) and CNPs treated group (group 3).

On the other hand, the use of salinity and nanoparticle treatment together significantly improved the levels of GPX and GR as compared to the other three groups (p<0.05). Concerning APX, salinity alone induced a significant impact on APX enzyme as compared to the control group (p<0.05), as well as CNPs treatment significantly elevated the levels of APX enzyme as compared to control and mild salinity stressed groups (p<0.05). Furthermore, the synergism between salinity and CNPs seed priming was apparent here, as this fourth group displayed a significantly higher APX value than the other three groups (p<0.05).

**Figure 5:**
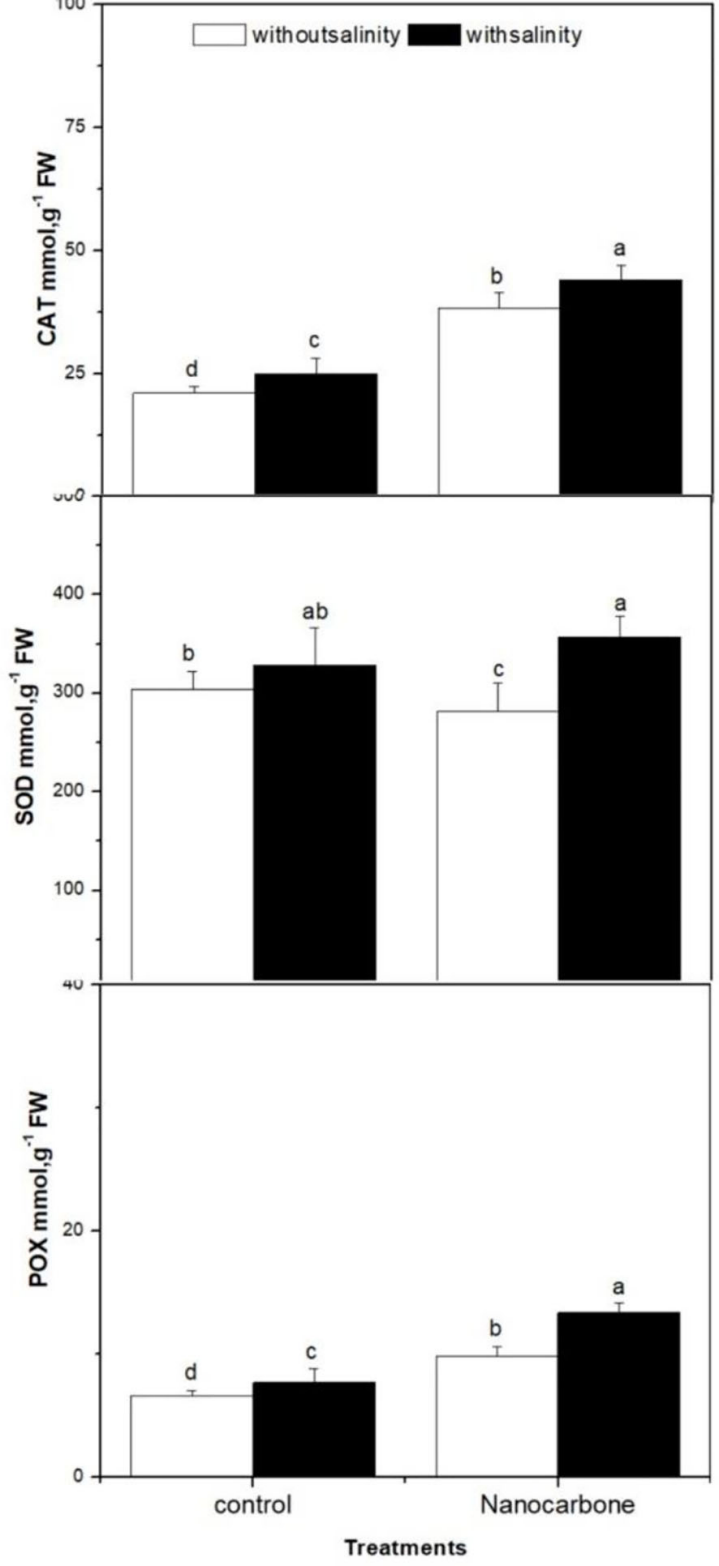
Effect of seed priming with carbon nanoparticles (CNPs) either alone or in combination with mild salinity on the production of antioxidant enzymes (CAT, SOD and POX) by radish sprouts. Data are represented by the means of three replicates and error bars represent standard error. Different small letters on the bars indicate significant differences between means at p<0.05.

Other important antioxidant enzymes were monitored in radish sprouts in response to mild salinity with or without seed priming using CNPs were CAT, SOD and POX (**Figure 5**). CAT enzyme was significantly improved by mild salinity of sprouts when compared to the untreated control group (p<0.05). Besides, the CNPs treated group had a significantly higher level of CAT enzyme as compared to the control group and group treated with mild salinity alone (p<0.05). Surprisingly, seed priming with CNPs duplicated the values of CAT enzyme when compared to the control group (p<0.05), and its impact was significantly greater than salinity. A similar scenario was noticeable in the case of POX enzyme, however, the effects of mild salinity and CNPs individually or combined on POX enzyme were not as significant as in CAT enzyme. Regarding SOD enzyme, mild salinity and CNPs treatment when applied separately did not induce the levels of that enzyme, conversely, when both treatments were applied combined significantly enhanced the values of SOD enzyme (p<0.05).

## 4 Discussion

Salinized land is progressively growing around the world. In order to fulfill the need of the growing population, the food supply is put at risk by salt stress, which significantly affects agricultural productivity and quality (Li et al., 2022). Salinity induces a variety of responses in plants and algae including morphological, biochemical, physiological, and molecular changes. It causes an ionic imbalance that results in toxicity, osmotic stress, and the generation of ROS (Hassan et al., 2020). Yet, mild salinity was deliberately applied and showed a favorable impact on plant resistance and antioxidant activity (Galli et al., 2016). In the current study, we applied mild salinity along with CNPs priming to improve the sprouting of radish seeds, as well as their nutritional composition and antioxidant resistance. The obtained results showed that mild salinity significantly decreased the fresh and dry weights of radish sprouts in comparison with the control group. It was reported that the radish was sensitive to salinity, besides, seed germination is the first delicate phase of a plant’s life cycle (Ahmad and Sharma, 2008). Furthermore, salt stress can also negatively impact seed germination and seedling growth in radish plants. The same result was observed by (Roy et al., 2022) who also demonstrated that the suppression of germination might be mainly due to osmotic stress and salinity also decreased germination percentage. Similar results revealed that the dry weights (DW) of roots and leaves of maize reduced linearly with the increase in salinity. This result may indicate that lowered growth in roots and lowered CO_2_ assimilation were correlated to salinity. The linear relationship between growth inhibition in these organs and rising Na+ buildup inside tissues is also present. Stomatal closure and/or damage to the photosynthetic apparatus caused by the buildup of Na+ and Cl- ions in leaf tissues may have reduced CO_2_ uptake, stunted development, and the accumulation of DW. (Abdelgawad et al., 2016). Additionally, it has previously been discovered that decreased DW and Na+ ion buildup in root and shoot tissues are related. The root and shoot length, FW and DW of ground nut (*Vigna subterranean*) decreased in correlation to increasing salinity (Ambede et al., 2012). On the other hand, (Cogo et al., 2011) found that strawberry plants treated with mild salinity (40 mM NaCl) showed statistically higher fresh weight than those exposed to high salinity stress (80 mM NaCl), suggesting that the mild stress resulted in beneficial effects for leaf production, a result that could be of interest in the production of leafy vegetables. Similar findings were seen for CO_2_ assimilation rates, where the group with mild salinity displayed significantly higher values (P 0.05) than the other groups, indicating higher photosynthetic rates. Salt stress often lowers photosynthetic rates in most plant species (Nawaz et al., 2010); however, improved photosynthetic rates resulting from mild salt stress were reported in *Periploca sepium* plants (Sun et al., 2011).

In order to mitigate the harmful effect of salinity on radish, seed priming with CNPs was applied. Seed priming is a significant, straightforward, profitable, and secure method because of its low environmental impact and potential benefits for agriculture (Joshi et al., 2020). In the present study, seed priming with CNPs significantly enhanced the fresh and dry weights of radish sprouts as compared with control in salinity-treated and untreated groups. Carbon nanomaterials priming has been shown to successfully increase germination and seedling stress tolerance and exhibited a respectable potential to improve plant stress tolerance (Raja et al., 2019).

In this regard, the safety and low toxicity of low concentrations of carbon nanomaterials to biological systems were previously reported by several studies (Mukherjee et al., 2016). Interestingly, carbon nanomaterials have gained the interest of many researchers for their unique physicochemical properties, such as high photostability, good biological compatibility, and low toxicity; these characteristics make them of promising potential applications in bioimaging, sensing, and agriculture (Hurt et al., 2006; Cao et al., 2007; Su et al., 2018). It was reported that the effect of carbon nanotubes on plant is concentration dependent. Low concentrations of carbon nanotubes were more beneficial to plants than higher concentrations. As they induced a reverse effect at high concentrations (Srivastava et al., 2015). Additionally, the cytotoxicity of carbon nanomaterials was previously measured by Su et al. (2018). It was found that A549 cells (lung cancer cell lines) showed 93% viability after incubation with carbon nanomaterials for 48 h even at a high concentration of 500 µg/mL, which suggests that carbon nanomaterials have a good biocompatibility and very low cytotoxicity even at high concentrations.

Interestingly, the application of CNPs through seed priming seemed to increase growth parameters and photosynthetic pigments with or without mild salinity in radish sprouts. In fact, this approach showed CNPs uptake and internalization into plant cells, leading to various interactions between plants and CNPs that can result in subtle to pronounced alterations in biochemical, physiological, and biological features as well as plant genetics (González-García et al., 2022). Our results were consistent with previous studies on sweet basil (Gohari et al., 2020) and wheat (T*. aetivum*) (Xiao et al., 2019). Many studies revealed that the increase in growth and photosynthetic parameters could be owing to the increase of the expression of genes that control cell division and cell wall extension as well as upregulating the genes that code for the protein aquaporin and aquaporin proteins. CNPs can also cause the creation of water channels (aquaporins) in seed coats which ended in faster plant growth, greater productivity, and better ability to withstand salt stress (Hatami et al., 2017; Bárzana et al., 2022).

Remarkably, the outcomes of the present study showed that the CNPs priming could mitigate the salt effect on growth and photosynthesis parameters by improving the accumulation of rubisco activity and α-carotene, β-carotene, lutein as well as β-cryptoxanthin. It was discovered that CNPs have the ability to internalize into chloroplasts, alter their structure, and expand their size. This has an advantageous impact on the synthesis of photosynthetic pigments like chlorophyll and accessory pigments like carotenoids and lycopene (Zhao et al., 2022). This is crucial for seed priming because, in addition to being essential for autotrophic development, chloroplasts are also required for seed germination and their activity can be essential in the presence of abiotic stress (Chen et al., 2021). The efficiency of photosynthesis can be increased by CNPs, as well as other critical biological processes like cell proliferation, cytoskeletal redox processes, and stress responses related to chloroplast development and protection (Han et al., 2022).

While analyzing the impact of CNPs on radish sprouts’ salt tolerance, in the present study, we also observed that seed pretreatment with CNPs remediated salt stress in radish sprouts *via* enhancing amino acids and soluble sugar contents. It was obvious that salinity affected the activity of enzymes involved in the biosynthesis of sugars causing a decline in sucrose, proline, P5CS, PRODH, sucrose P synthase, and invertase. Increased proline and sugar content was used as a marker for assisted selection to improve salinity and tolerance (Wu et al., 2013). The CNPs priming improved the accumulation of both osmolytes of radish sprouts under salt stress, therefore the recovered the osmoprotectant effect of proline and sugar accumulation.

Under conditions of mild salinity, the mechanisms of adaptation to stress are the production of antioxidant compounds and increased activity of the enzymes related. In this investigation, CNPs priming showed a potential to be used as elicitors or biostimulants for the induction of bioactive compounds in radish sprouts since they positively modify the expression of genes involved in the biosynthesis of secondary metabolites (González-García et al., 2022). These metabolites are also capable of mitigating the limitations associated with mild salinity. In fact, the results showed that by priming with CNPs, salt tolerance can be induced in radish sprouts against salinity by activating the plant defense system through the biosynthesis of polyphenols, flavonoids, and anthocyanin metabolites. A synergistic effect between CNPs priming and mild salinity on polyphenols and anthocyanin metabolites was noticeable. Some previous studies suggest that nanoparticles may directly interact with the enzymes involved in anthocyanin biosynthesis, leading to an increase in the rate of pigment production. More research is needed to fully understand the effects of CNPs on anthocyanin biosynthesis and the potential implications for plant growth and development. Similar results were reported by Hashemi et al. (Hashemi et al., 2019) who found that nanoparticle treatment increased anthocyanin production in soybean. Furthermore, in the present study, CNPs priming weakened the effect of salt stress on other antioxidant metabolites including ascorbates (ASC), glutathione (GSH), and tocopherols. Besides, there was a synergistic effect between mild salinity and CNPs on the levels of these antioxidant metabolites. In this regard, it was reported that although extreme abiotic stresses, like salt stress, have a negative impact on plant growth, mild stresses can be purposefully induced to increase the antioxidant content of the plant’s edible section and promote plant tolerance to stressful circumstances (Kim et al., 2008; Cogo et al., 2011).

Due to the rise in reactive nitrogen species (ROS) generation brought on by salt stress, it has a detrimental effect on a number of biochemical and physiological processes. In this regard, several authors have reported the use of CNPs in seed priming and in the induction of tolerance to environmental stresses by increasing the enzymatic activity of CAT, SOD, APX, and GPX as well as other bioactive substances including ascorbic acid and GSH under salt stress (Soltabayeva et al., 2021). In this present study, the CNPs priming in combination with mild salinity improved the activity of APX, GPX, GR as well as CAT, SOD, and POX in radish sprouts.

In conclusion, it was obvious that CNPs priming of seeds helped radish sprouts to exhibit a significant mechanism of adaptation to mild salinity by producing different antioxidant metabolites and enzymes to prevent the oxidation of other molecules by inhibiting the initiation and elongation of the oxidative chain reaction of ROS. The seed priming with CNPs has the potential to be a significant elicitor or biostimulant for the induction of bioactive compounds in radish sprouts which mitigates the limitations associated with mild salinity.

## Data availability statement

The original contributions presented in the study are included in the article/supplementary material. Further inquiries can be directed to the corresponding author.

## Author contributions

AH, AZ, FA, MB, and HA conceived the study. FA, AZ, RH, AR, AH, and HA conducted the experiments. AZ, AH, MB and HA analyzed the results and performed the statistics and illustrations. FA, and RH wrote the manuscript draft. All authors contributed to the article and approved the submitted version.
